# First-Line *Helicobacter pylori* Eradication with Vonoprazan, Clarithromycin, and Metronidazole in Patients Allergic to Penicillin

**DOI:** 10.1155/2017/2019802

**Published:** 2017-10-18

**Authors:** Soichiro Sue, Nobumi Suzuki, Wataru Shibata, Tomohiko Sasaki, Hiroaki Yamada, Hiroaki Kaneko, Toshihide Tamura, Tomohiro Ishii, Masaaki Kondo, Shin Maeda

**Affiliations:** ^1^Department of Gastroenterology, Yokohama City University Graduate School of Medicine, Yokohama, Japan; ^2^Department of Gastroenterology, Institute for Adult Diseases, Asahi Life Foundation, Tokyo, Japan; ^3^Advanced Medical Research Center, Yokohama City University, Yokohama, Japan

## Abstract

**Aim:**

To assess the efficacy of 7-day first-line *Helicobacter pylori* eradication with vonoprazan (VPZ), clarithromycin (CAM), and metronidazole (MNZ) in patients with penicillin allergy.

**Methods:**

Patients with penicillin allergy, diagnosed with *Helicobacter pylori* infection and did not have history of *Helicobacter pylori* eradication, were eligible for the study. Twenty patients were prospectively treated with 20 mg VPZ twice daily, 200 or 400 mg CAM twice daily, and 250 mg MNZ twice daily for 7 days. We also collected the data from 30 patients retrospectively treated with proton pump inhibitor (PPI), CAM, and MNZ. Safety was evaluated in patients completing an adverse effect questionnaire.

**Results:**

Both the intention-to-treat and per-protocol effectiveness of VPZ-based eradication were 100% (95% CI: 86.1–100%; *n* = 20). The eradication rates of PPI-based regimen were 83.3% (95% CI: 65.3–94.4%) in the ITT and 82.7% (95% CI: 64.2–94.2%) in the PP analyses. Abdominal fullness was more frequent in VCM compared to PCM. However, all patients with VCM regimen had taken 100% of their course of medication.

**Conclusion:**

Triple therapy with VPZ, CAM, and MNZ is well tolerated and effective for eradicating *Helicobacter pylori* in patients allergic to penicillin. This study was registered in the UMIN Clinical Trials Registry as UMIN000016335.

## 1. Introduction

A recent systematic review and meta-analysis showed that *Helicobacter pylori* eradication reduces the incidence of gastric cancer irrespective of baseline risk [1]. A 40% reduction in the risk of gastric cancer would increase to 75%, if the eradication resulted complete and sustained [2, 3]. Thus, *H*. *pylori* eradication regimens with excellent eradication rates (ERs) (≥90–95%) should be prescribed [4, 5]. In patients who are allergic to penicillin, regimens without amoxicillin (AMPC) are used for *H*. *pylori* eradication. The recent Maastricht V/Florence Consensus Report stated that a proton pump inhibitor (PPI)/clarithromycin (CAM)/metronidazole (MNZ) combination (PCM regimen) may be prescribed in areas with low rates of CAM resistance, such as Southeast Asia. In areas with high rates of CAM resistance, the PCM regimen has an unacceptable ER of less than 80% [6, 7]. In such cases, PPI-tetracycline-MNZ [8], bismuth-PPI-tetracycline-MNZ (bismuth-based quadruple therapy, BQT) [9], bismuth-PPI-tetracycline-furazolidone (modified BQT) [10], and PPI-sitafloxacin- (STFX-) MNZ [11] regimens are effective. A recent study revealed that the BQT regimen is effective for cases of CAM and MNZ resistance, but results in higher adverse event rates compared to 14-day triple therapy (67% [358/533, 95% CI: 63–71] versus 47% [252/535, 95% CI: 43–51]) [12]. Other regimens are also associated with more adverse events than the PCM regimen. Thus, the optimum regimen for patients with penicillin allergy must have an excellent ER and a safety profile identical or superior to that of the PCM regimen.

Vonoprazan (VPZ), the first of a novel class of acid suppressants (potassium-competitive acid blockers, P-CABs), was approved for *H*. *pylori* eradication in Japan in February 2015. In a phase III, randomized, double-blind study, the VPZ/AMPC/CAM ER of 92.6% (*n* = 324) was noninferior to that of lansoprazole (LPZ, PPI)/AMPC/CAM (75.9%; *n* = 320; *p* < 0.001) [13]. A meta-analysis showed a similar efficacy for PPI/AMPC/CAM: 81% (95% CI: 79–83%) in intention-to-treat (ITT) and 84% (82–86%) in per-protocol (PP) analyses and PPI/CAM/MNZ: 81% (78–83%) in ITT and 84% (82–86%) in PP analyses [14]. In a subgroup analysis of a CAM-resistant subpopulation in a VPZ phase III study, the VPZ/AMPC/CAM ER of 82.0% (*n* = 100) was significantly higher (*p* < 0.0001) than the LPZ/AMCP/CAM ER of 40.0% (*n* = 115) [13]. Therefore, the VPZ/CAM/MNZ regimen may be useful for patients with penicillin allergy. In the current study, we assessed the efficacy and safety of the VPZ/CAM/MNZ regimen as a first-line *H*. *pylori* eradication therapy for patients allergic to penicillin.

## 2. Materials and Methods

### 2.1. Study Design

This was a first prospective and registered study of the efficacy and safety of a 7-day first-line *H*. *pylori* eradication regimen (VPZ/CAM/MNZ [VCM]) in patients with a documented allergy to penicillin. The protocol and informed consent forms were reviewed and approved by the Ethics Committee of Yokohama City University Hospital. This study was registered in the UMIN Clinical Trials Registry as UMIN000016335. After the approval of protocol and registration, this study was performed prospectively with written informed consent and Adverse Effects Questionnaires (later in detail) were filled by patients during therapy. We also collected retrospective data from our previous study of a 7-day PPI (LPZ) or esomeprazole (ESO/CAM/MNZ [PCM]) regimen in patients with penicillin allergy for comparison. The design of comparison between prospective VCM data and retrospective PCM data was approved and registered. It is important to note that PCM in a previous study was also conducted with written informed consent and by answering the Adverse Effects Questionnaire, and we used them in this study as retrospective data. The study was conducted in Yokohama City University (YCU) Hospital (Kanagawa, Japan) and the Institute for Adult Diseases, Asahi Life Foundation in Tokyo, Japan (Asahi Hospital). After approval of VPZ, the VPZ/CAM/MNZ regimen was used, whereas before approval, the PPI/CAM/MNZ regimen was used. This study is registered at https://upload.umin.ac.jp/cgi-open-bin/ctr_e/ctr_view.cgi?recptno=R000018955. The registration identification number is UMIN000016335. This trial registry (http://www.umin.ac.jp/ctr/index/htm) is accepted by the International Committee of Medical Journal Editors (ICMJE).

### 2.2. Participants

Male or female *H*. *pylori*-positive patients aged ≥ 20 years with a documented allergy to penicillin were eligible for inclusion. Penicillin allergy was diagnosed by physicians as being allergic to past penicillin derivatives. Subjects with any of the following were excluded: history of *H*. *pylori* eradication therapy; pregnancy or lactation; history of allergy to the drugs used (CAM and MNZ); severe liver dysfunction; severe renal dysfunction; severe heart dysfunction; and disqualification by their physicians. All of the eligible subjects were treated with the VCM regimen, including CAM-resistant *H*. *pylori*-infected patients.

### 2.3. Determination of *H*. *pylori* Status

*H*. *pylori* status was determined by detection of anti-*H*. *pylori* immunoglobulin G (HpIgG), a rapid urease test (RUT), culture, pathology (histology), or a carbon 13-labeled urea breath test (^13^C-UBT). *H*. *pylori* eradication was primarily determined by a UBT with 100 mg UBT tablets (Otsuka Pharmaceutical Co. Ltd., Tokyo, Japan) using a cutoff of 2.5‰ or, in a few cases, with a stool *H*. *pylori* antigen test, both of which are considered standards [15, 16]. For all of the participants, a follow-up UBT was performed after at least 4 weeks, and typically over 7 weeks, after completion of treatment to confirm successful eradication: 8.06 ± 2.39 weeks in VCM, 11.23 ± 4.83 weeks in PCM, and 9.96 ± 4.28 weeks in total. There is not a significant difference between VCM and PCM (*p* = 0.129). All subjects were asked to stop taking PPIs or VPZ from completion of treatment until the UBT. The UBT was performed by an external clinical inspection agency in all cases.

### 2.4. Treatment

We analyzed first-line triple therapy with 20 mg bid VPZ in combination with 200 or 400 mg bid CAM plus 250 mg bid MNZ bid for 1 week (VCM regimen) (Table 1). We also collected data on first-line triple therapy with a PPI (30 mg bid LPZ or 20 mg bid ESO) in combination with 200 or 400 mg bid CAM plus 750 mg bid MNZ for 1 week (PCM regimen) (Table 1). Because CAM does not affect the *H*. *pylori* rate [13], the dose of 200 mg bid CAM was used in the majority of cases. A previous meta-analysis showed that the PPIs and doses used in this study (20 mg bid ESO and 30 mg bid LPZ) do not affect the ER [17]. All of the treatments were administered orally, and the subjects were followed for at least 4 weeks and were evaluated for *H*. *pylori* status.

### 2.5. Procedures

After study participation, a physician completed the study registration form, which included sex, age, endoscopic findings, method of diagnosis of *H*. *pylori* infection, eradication regimen (including dose of CAM), determination method of *H*. *pylori* infection, and start date of eradication therapy. After eradication therapy, *H*. *pylori* eradication was assessed by a UBT (stool *H*. *pylori* antigen test with immune-chromatography kit (Wakamoto Co. Ltd, Tokyo, Japan) was also used in PCM regimen). Then, a case report form was completed that included the date of the eradication assessment, compliance with treatment, adverse events, and confirmation of the washout of acid suppressants after eradication. In this study, an Adverse Effects Questionnaire (AEQ) was completed by patients during therapy and collected at the visit after eradication therapy in all cases. The AEQ contained 13 questions (diarrhea, dysgeusia, nausea, anorexia, abdominal pain, heartburn, urticaria, headache, abdominal fullness, eructation, vomiting, fatigue, and others), and patients selected from among the following subjective responses: none (AEQ 0), weak (AEQ 1), moderate (AEQ 2), or strong (AEQ 3). The primary end-point was the *H*. *pylori* ER of the VCM regimen in patients with a documented allergy to penicillin. The secondary end-point was safety, as evaluated by the AEQ.

### 2.6. Statistical Analysis

Categorical data were compared using the Fisher's exact test. All of the *p* values were two tailed, with the level of statistical significance set at 0.05. Statistical analyses were performed using SPSS software (ver. 24).

## 3. Results

**Figure 1:**
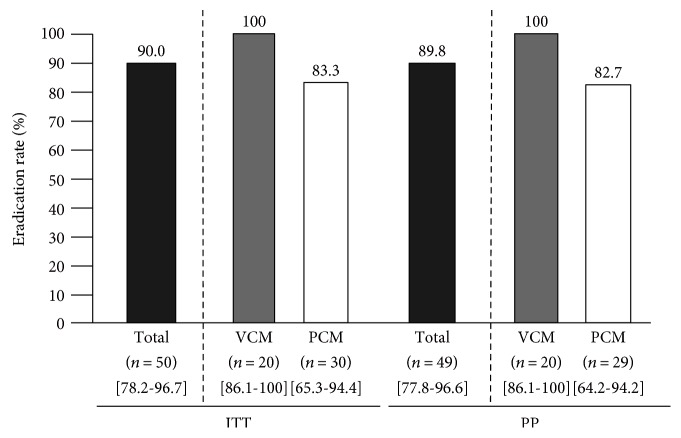
Eradication rates of VCM: vonoprazan/CAM/MNZ 1-week eradication therapy and PCM: PPI (LPZ or ESO)/CAM/MNZ 1-week eradication therapy. ITT: intention-to-treat analyses; PP: per-protocol analyses; CAM: clarithromycin; MNZ: metronidazole; vonoprazan: a novel class of acid suppressants (potassium-competitive acid blockers (P-CABs)); PPI: proton pump inhibitor; : range of 95% confidence interval.

A total of 50 patients with penicillin allergy were enrolled. The ERs of VPZ or PPI, CAM, and MNZ (VCM or PCM) in ITT and PP analyses were 90.0% (95% CI: 78.2–96.7; *n* = 50) and 89.8% (95% CI: 77.8–96.6; *n* = 49), respectively (Figure 1). All patients with VCM regimen were prospectively enrolled from February 2015 to April 2016. Because most of patients registered to this study in our hospital were visited with referral letter for eradication with penicillin allergy, the accurate number or rate with penicillin allergy in all *H. pylori*-infected patients is not clear. As shown in Table 1, the mean age of the patients was 69.0 ± 10.2 years, and 20% were male. All 20 patients took the full course of medication and underwent the UBT test at 8.06 ± 2.39 weeks after drug withdrawal (range, 5.0 to 15 weeks). No patients failed to return for follow-up. A dose of 400 mg CAM per day was administered in 16 cases (80.0%). Endoscopic findings were mostly gastritis (gastroduodenal ulcer, gastric cancer, and MALT; one patient each), and *H*. *pylori* infection was diagnosed by HPIgG, culture, RUT, UBT, pathology, urine test, or stool antigen test. Successful eradication was achieved in all of the cases by the VCM regimen, for an ER of 100% (95% CI: 86.1–100.0%) by both ITT and PP analyses.

We also retrospectively evaluated 30 patients with a penicillin allergy in whom *H*. *pylori* eradication using first-line PCM therapy was successful. The PPIs used were LPZ (*n* = 20) and ESO (*n* = 10). Patient characteristics are shown in Table 1, and the drug withdrawal period was 11.2 ± 4.5 weeks. Successful eradication was achieved in 25 cases, while in 1 case, poor compliance (64% of the course of medication completed) was observed because of marked dysgeusia, anorexia, urticarial, and fatigue. Eradication was also successful in this case. The ERs of the PCM regimen were 83.3% (95% CI: 65.3–94.4%) in the ITT and 82.7% (95% CI: 64.2–94.2%) in the PP (Figure 1) analyses.

The frequencies of adverse effects during therapy, as assessed by AEQ, are shown in Table 2. In 15% of cases, AEQ 3 abdominal fullness was reported. In 10% of cases, AEQ 3 nausea was reported. AEQ 3 anorexia, abdominal pain, and headache were each experienced in 5% of the cases. In terms of AEQ 2 or 3 adverse reactions, abdominal fullness was experienced in 30% cases; dysgeusia, nausea, abdominal pain, and fatigue were in 15% cases; anorexia, heart burn, and headache were in 10% cases; and diarrhea, belch, and mouth discomfort (others) were in 5% cases. There were no differences between VCM and PCM in AEQ 3. Only abdominal fullness in AEQ 2 or 3 was more frequent in VCM compared to PCM. However, all of the patients with VCM regimen had taken 100% of their course of medication.

## 4. Discussion

This study assessed the efficacy and safety of 7-day VCM therapy in patients with penicillin allergy. The 100% success (95% CI: 86.1–100%) and 100% compliance indicate this novel regimen's possibility for excellent (95–100%) grading defined by Graham et al. [5]. In addition, the ER of the VCM regimen was 16.7% (95% CI: 3.3–30%) higher than that of the PCM and was similar to the 16.7% (95% CI: 11.2–22.1%) (VAC 92.6% versus LAC 75.9%) in the VPZ phase III study [13].

Our result is in agreement with a previous report of the superiority of VPZ-based regimens in areas with a high rate of CAM resistance. The first-line ER of a VPZ/AMPC/CAM (VAC) regimen in a CAM-resistant population (82%, *n* = 100) was higher than that of a PPI- (LPZ-) based regimen (40%) (*n* = 115) (*p* < 0.0001). We have confirmed this in real clinical practice that VAC exhibited an ER of 73.2% (*n* = 56) in a CAM-resistant population [18]. These results differ from those of PPI-based regimens; CAM resistance reduced the effectiveness to 55% (95% CI: 33–78%) according to a meta-analysis [19]. This study was conducted in areas of high CAM resistance; the *H*. *pylori* CAM resistance rate was ~40% in YCU and was an average of 26% in hospitals in the Kanagawa area. Thus, the VCM regimen can be used in areas of low and high rates of CAM resistance, including Japan.

The abovementioned result was expected based on the novel mechanism of action of VPZ: K^+^-competitive and reversible activity [20]. This results in rapid achievement of maximal efficacy (2-3 h for VPZ versus 3–5 days for PPIs), long-lasting effects (VPZ, dose-dependent accumulation in parietal cells; PPIs, unstable under acidic conditions and dependent on blood levels), and low rates of polymorphism (VPZ, CYP3A4; PPIs, and CYP2C19) [21]. In contrast, relatively poor (81–84%) result of the comparison arm of PCM: 83.3% (65.3–94.4%, *n* = 30) is reasonable from previous meta-analysis: 81% (95% CI: 78% to 83%, ITT analysis) [14]. In addition, CAM and AMPC function in the growth phase [22] and *H*. *pylori* grows optimally at pH > 5; thus, these features of VPZ explain the excellent results of VCM and VAC.

The first clinical implication of this study is the use of VCM instead of a 7-day PPI/MNZ/STFX regimen (PMS) in Japan, which was recently used for patients allergic to penicillin and showed an excellent ER of 100% (95% CI: 86.1–100.0%, *n* = 19) [11]. PMS as a third-line regimen also shows good efficacy (90.9%; 95% CI: 78.3–97.5%; *n* = 44) [23]. However, diarrhea (21.4% in the first-line study and 32.0% in the third-line study) and loose stool (35.7% in the first-line study and 68% in the third-line study) were reported as major adverse events, which were in higher rates than those of VCM (5% moderate AEQ and no severe diarrhea according to AEQ score).

The second clinical implication is the possibility of VPZ-based concomitant therapy and a bismuth-based VCM regimen [24, 25]. Both this study and the VPZ phase III study [13] suggest the utility of VPZ-based regimens in CAM-resistant populations.

Our results must be interpreted with the following limitations in mind. First, the sample size was small and study design was not RCT. However, 3–7% of patients are allergic to penicillin in Japan [26] and elsewhere [27]; therefore, a large-scale study with VCM regimen is difficult in a limited period of time after approval of VPZ. Second, we could not assess resistance to CAM and MNZ in the majority of cases (10/13). One case had the following minimum inhibitory concentration values: CAM 16 mg/L, AMPC 0.5 mg/L, STFX 0.25 mg/L, MNZ 4 mg/L, but eradication was successful in this patient. The other two cases were susceptible (AMPC < 0.03, CAM < 0.03, STFX 0.06, and MNZ 2; AMPC < 0.03, CAM < 0.03, STFX < 0.03, and MNZ 2); these patients also experienced successful eradication. Further study of VCM regimen with CAM and MNZ resistance information in all cases is needed.

During manuscript preparation, a similar study was published. This reported an ER of the VCM regimen in patients with penicillin allergy of 92.9%. The authors also suggested that VCM could be used in such patients [28]. Our study is important, because our study is the first prospective as well as registered study of VCM regimen, and our study used the same Adverse Effect Questionnaire, which is available to be compared with PCM.

## 5. Conclusions

Our data demonstrated that 7-day VCM therapy has an excellent ER and safety profile in patients with a penicillin allergy in areas of a high rate of CAM resistance.

## Figures and Tables

**Table 1:** Patient backgrounds.

	VCM	PCM
Age	69.0 ± 10.2	66.5 ± 8.5
Male, %	20.0	53.3
CAM 200 bid, %	80.0	96.7
Evaluation by UBT, %	100	93.3
*Endoscopic findings*, %		
Gastroduodenal ulcer	10.0	40.0
Gastric cancer	5.0	3.3
Gastric adenoma	0	3.3
MALT	5.0	0
Gastritis only	80.0	53.3
*Diagnosis of infection*		
HpIgG	30.0	43.3
RUT	20.0	20.0
Culture	25.0	3.3
Pathology	5.0	30.0
UBT	10.0	3.3
Urine, stool antigen	10.0	0

VCM: vonoprazan/CAM/MNZ 1-week eradication therapy; PCM: PPI (LPZ or ESO)/CAM/MNZ 1-week eradication therapy; CAM 200 bid, %: percentage of CAM 200 mg twice per day (400 mg/day) against CAM 400 mg twice per day (800 mg/day); evaluation by UBT, %: percentage determined by ^13^C-urea breath test versus *H. pylori* stool antigen test; endoscopic findings: all participants underwent endoscopy before eradication therapy; RUT: rapid urease test; UBT: ^13^C-urea breath test.

**Table 2:** Safety of VCM versus that of PCM by questionnaire.

	Any (AEQ 2 or 3)			AEQ 3		
	VCM	PCM	*p*	VCM	PCM	*p*
Diarrhea	5.0%	6.7%	1	0%	0%	1
Dysgeusia	0%	6.7%	0.38	0%	6.7%	0.51
Nausea	15.0%	0%	0.06	10.0%	0%	0.16
Anorexia	10.0%	3.3%	0.56	5.0%	3.3%	1
Abdominal pain	15.0%	3.3%	0.29	5.0	0%	0.40
Heart burn	10.0%	6.7%	1	0%	3.3%	1
Hives	0%	3.3%	1	0%	3.3%	1
Headache	10.0%	0%	0.16	5.0%	0%	0.40
*Abdominal fullness*	**30.0%**	**3.3%**	**0.012**	**15.0%**	0%	0.06
Belch	5.0%	0%	0.40	0%	0%	1
Vomiting	0%	0%	1	0%	0%	1
General malaise	15.0%	3.3%	0.29	0%	3.3%	1
Others	5.0%	3.3%	1	0%	0%	1

AEQ: adverse effects questionnaire; AEQ 2: moderate; AEQ 3: strong, VCM: vonoprazan/CAM/MNZ 1-week eradication therapy; PCM: PPI (LPZ or ESO)/CAM/MNZ 1-week eradication therapy.
